# Gene–Nutrient Interactions in Obesity: *COBLL1* Genetic Variants Interact with Dietary Fat Intake to Modulate the Incidence of Obesity

**DOI:** 10.3390/ijms24043758

**Published:** 2023-02-13

**Authors:** Junkyung Kwak, Dayeon Shin

**Affiliations:** Department of Food and Nutrition, Inha University, Incheon 22212, Republic of Korea

**Keywords:** *COBLL1*, rs6717858, obesity, dietary fat, Korean genome and epidemiology study

## Abstract

The *COBLL1* gene is associated with leptin, a hormone important for appetite and weight maintenance. Dietary fat is a significant factor in obesity. This study aimed to determine the association between *COBLL1* gene, dietary fat, and incidence of obesity. Data from the Korean Genome and Epidemiology Study were used, and 3055 Korean adults aged ≥ 40 years were included. Obesity was defined as a body mass index ≥ 25 kg/m^2^. Patients with obesity at baseline were excluded. The effects of the *COBLL1* rs6717858 genotypes and dietary fat on incidence of obesity were evaluated using multivariable Cox proportional hazard models. During an average follow-up period of 9.2 years, 627 obesity cases were documented. In men, the hazard ratio (HR) for obesity was higher in CT, CC carriers (minor allele carriers) in the highest tertile of dietary fat intake than for men with TT carriers in the lowest tertile of dietary fat intake (Model 1: HR: 1.66, 95% confidence interval [CI]: 1.07–2.58; Model 2: HR: 1.63, 95% CI: 1.04–2.56). In women, the HR for obesity was higher in TT carriers in the highest tertile of dietary fat intake than for women with TT carriers in the lowest tertile of dietary fat intake (Model 1: HR: 1.49, 95% CI: 1.08–2.06; Model 2: HR: 1.53, 95% CI: 1.10–2.13). *COBLL1* genetic variants and dietary fat intake had different sex-dependent effects in obesity. These results imply that a low-fat diet may protect against the effects of *COBLL1* genetic variants on future obesity risk.

## 1. Introduction

The prevalence of obesity is steadily increasing worldwide [1]. Obesity is a major health issue that causes many complications, including mental illness, cardiovascular disease (CVD), metabolic syndrome, cancer, and even death [1]. According to a study comparing chronic disease prevalence before and after the coronavirus disease 2019 (COVID-19) pandemic using data from the 2011 to 2020 National Health and Nutrition Examination Survey (NHANES), the prevalence of obesity in 2017–2019 was 34.2%, and that in 2020 was 38.4%; an increase of 4.2% was observed in Korean adults [2]. In middle-aged men aged 50–59 years, the prevalence of obesity was 42.8% in 2017–2019 and 48.3% in 2020, an increase of 5.4% [2]. In middle-aged women aged 50–59 years, the prevalence of obesity was 30.2% in 2017–2019 and 32.4% in 2020, an increase of 2.2% [2]. Overweight and obesity in middle aged individuals are risk factors for Alzheimer’s disease in the elderly [3]. Obesity in middle-aged individuals may precede dementia as a vascular risk factor and decreased neuroprotective effects of leptin due to obesity contribute to Alzheimer’s disease [3]. In 2020, the prevalence of hypercholesterolemia, hypertension, and diabetes increased by 1.7%, 0.4%, and 1.4%, respectively, compared to that in 2017–2019 [2]. The prevalence of obesity has increased rapidly since the COVID-19 pandemic compared to its prevalence as associated with other chronic diseases [2]. This suggests that obesity is a consequence of the recent COVID-19 pandemic and environmental factors, including increased stress, reduced physical activity, and dietary changes [4,5]. Genetic factors also significantly influence obesity risk [6]. Approximately 3% of severely obese patients are genetically deficient in leptin and leptin receptors [7]. Leptin and leptin receptor deficiency lead to overeating, impaired satiety, and increased body fat due to difficulties in energy intake regulation [8,9].

Dietary fat contributes to obesity risk [10]. In the 1991–2015 China Health and Nutrition Survey, dietary fat was positively linked to overweight and obesity [11]. In addition, the ratio of dietary fat (kcal/day) to total energy intake was positively linked to obesity [11]. A dietary-fat-restricted diet caused a greater reduction in fat mass than a dietary-carbohydrate-restricted diet in 19 obese adults in the United States [12]. In a 4-week experimental animal study, a high-fat (HF) diet caused an imbalance in intestinal microorganisms, which was associated with weight gain and fat development in 12-week-old male C57bl6/J mice [13]. In an 8-week experimental animal study, a HF diet increased obesity risk, adipose tissue, and plasma cholesterol along with changes in the intestinal microbiome in healthy female C57BL/6N mice [14]. In a 16-week experimental animal study, the HF diet significantly increased body weight, triglyceride (TG) and cholesterol levels, and insulin intolerance compared to those associated with a low-fat (LF) diet, which resulted in obesity and diabetes in 6-week-old C57BL/6 mice [15]. Reduced dietary fat intake induces increases leptin sensitivity, which aids in weight loss [16]. The dietary fat has been linked to increased obesity risk in humans and animals through body fat and weight gain, increased leptin sensitivity, promotion of intestinal microbial destruction, and accumulation of TG and cholesterol [11,12,13,14,15,16,17].

Not all types of dietary fat adversely affect leptin secretion and sensitivity [18]. A high-polyunsaturated fatty acid diet prevents obesity [18]. Leptin, a known weight control protein, inhibits appetite and increases energy consumption by providing energy storage signals from adipocytes to the central nervous system [19]. Additionally, leptin resistance indicates an increased susceptibility to diet-induced obesity [20].

Inconsistent findings regarding the contribution of dietary fat in obesity exist [21,22]. A 12-month randomized trial found no difference between an LF diet and body fat percentage and weight in 194 adult women [21]. In an 18-month randomized trial, involving 122 obese women, an LF diet caused weight loss after 6 months; however, weight returned to baseline after 18 months [22]. These findings suggest that dietary fat does not significantly affect weight and body fat reduction [21,22].

*COBLL1* (cordon-bleu WH2 repeat protein-like 1) gene is associated with neural tube formation [23], central obesity [24], fasting insulin [25], type 2 diabetes risk [26], blood lipids [27], CVD risk [28], gastric cancer [29], prostate cancer [30], and leukemia [31,32]. *COBLL1* rs6717858 is associated with plasma leptin levels secreted from adipose tissue and obesity-related factors, such as body mass index (BMI), central obesity, waist circumference (WC), fat mass, and lipid levels (prominent effect in women) [26,27,33,34,35]. Decreased *COBLL1* expression is associated with adipose tissue dysfunction [36,37]. Genetic studies related to leptin circulation have identified a leptin-decreasing allele linked to higher fat mass, BMI, and obesity in adults [38,39]. The leptin increasing allele of *COBLL1/GRB14* (rs6738627) is strongly associated with body fat ratio, and the *COBLL1* gene is upregulated by a HF diet [40]. Decreased *COBLL1* expression affects obesity and dyslipidemia risk by increasing lipid storage in adipose tissue [36].

Several studies have examined the individual effects of the *COBLL1* gene and dietary fat intake in obesity [11,12,13,14,15,16,17,26,27,33,34,35]. However, only few studies have investigated the interaction between dietary fat and the *COBLL1* gene in obesity. Therefore, this study aimed to examine the interaction between *COBLL1* genetic variants and dietary fat in obesity using urban–rural prospective cohort data.

## 2. Results

### 2.1. General Characteristics of Participants at Baseline

We documented 627 cases of obesity over an average period of 9.2 years. Table 1 shows the general characteristics, biomarkers, and dietary intake based on sex and obesity status (obese men, *n* = 312; non-obese men, *n* = 1228; obese women, *n* = 315; non-obese women, *n* = 1200). Regardless of sex, the obese group had higher BMI and WC than those of the non-obese group (all *p*-values < 0.0001). Among men, the obese group had lower mean age, metabolic equivalent of task (MET), resident in Ausung, high-density lipoprotein (HDL) cholesterol level, and carbohydrate intake and higher household income, alcohol consumption, TG, total energy, protein, and fat intake than those of the non-obese group (all *p*-values < 0.05). Among women, the obese group had higher average diastolic blood pressure than that of the non-obese group (*p* < 0.05).

Table 2 shows the general characteristics, biomarkers, and dietary intake based on sex and *COBLL1* rs6717858 genotypes (TT vs. CT or CC) (TT genotype in men, *n* = 1220; CT or CC genotype in men, *n* = 320; TT genotype in women, *n* = 1253; CT or CC genotype in women, *n* = 262). HDL-cholesterol was higher in men with the CT or CC genotype than in men with the TT genotype (*p* = 0.03; 45.0 ± 10.2 vs. 46.5 ± 10.6). TG was lower in men with the CT or CC genotype than in men with the TT genotype (*p* = 0.0027; 162.0 ± 114.9 vs. 145.8 ± 76.2). The mean age was higher in women with the CT or CC genotype than in women with the TT genotype (*p* = 0.0082; 50.4 ± 8.8 vs. 52.0 ± 9.1). In women with the CT or CC genotype, average systolic blood pressure and diastolic blood pressure were higher than those in women with the TT genotype (all *p*-values = 0.01; 114.7 ± 18.0 vs. 117.8 ± 19.4; 74.9 ± 11.0 vs. 76.9 ± 12.1, respectively). In women with the TT genotype, fat intake was higher than that in women with the CT or CC genotype (*p* = 0.04; 14.2 ± 5.3 vs. 13.5 ± 5.5).

### 2.2. Association between Dietary Fat Intake and BMI

Table 3 shows the association between dietary fat intake (g/1000 kcal) and BMI. In men, there was no significant difference between dietary fat and BMI (beta ± standard error [SE]: −0.001 ± 0.009, *p* = 0.89 in Model 1; beta ± SE: −0.015 ± 0.009, *p* = 0.12 in Model 2). In women, there was no significant difference between dietary fat and BMI (beta ± SE: −0.004 ± 0.008, *p* = 0.58 in Model 1; beta ± SE: −0.003 ± 0.008, *p* = 0.71 in Model 2).

### 2.3. Association between Dietary Fat Intake and Incidence of Obesity

Table 4 shows the association between the tertiles of dietary fat intake (% energy) and incidence of obesity. The participants were divided into three groups according to dietary fat intake in men (Tertile 1: 10.7, 2.9–13.2; tertile 2: 15.4, 13.2–17.5; tertile 3: 20.3, 17.5–35.1) and women (Tertile 1: 8.9, 1.9–11.5; tertile 2: 13.7, 11.5–15.9; tertile 3: 18.9, 15.9–42.0).

In men, the highest fat intake was associated with a 41% higher incidence of obesity than that with the lowest fat intake in Model 1 (hazard ratio [HR]: 1.41, 95% confidence interval [CI]: 1.02–1.95). No significant difference in obesity was observed in Model 2 (HR: 1.37, 95% CI: 0.98–1.93). In women, no significant association with obesity was observed in tertile 3 (compared to that observed in tertile 1) (HR: 1.31, 95% CI: 0.97–1.78 in Model 1; HR: 1.35, 95% CI: 0.99–1.84 in Model 2).

We further investigated the association between dietary fat intake and obesity (Tables S1 and S2). The participants were divided according to the median dietary fat value (men: < 15.4 vs. ≥ 15.4, women: < 13.7 vs. ≥ 13.7; Table S1). Obesity and dietary fat did not differ significantly according to the median value. In men, a 1% increase in fat intake was associated with a 3% higher incidence of obesity in Model 1 (HR: 1.03, 95% CI: 1.01–1.05). In women, there was no significant difference in obesity as fat intake increased by 1%. Furthermore, the participants were divided according to dietary fat (% energy) (<15% vs. ≥15%; Table S2), and no significant differences were noted.

### 2.4. Association between COBLL1 rs6717858 Genotypes and Incidence of Obesity

Table 5 shows the association between the *COBLL1* rs6717858 genotypes and incidence of obesity. In men, there was no significant difference in obesity based on the CT or CC genotype (HR: 1.08, 95% CI: 0.83–1.41 in Model 1; HR: 1.08, 95% CI: 0.83–1.41 in Model 2). In women, there was no significant association between the incidence of obesity and CT or CC genotype (HR: 1.29, 95% CI: 0.97–1.71 in Model 1; HR: 1.27, 95% CI: 0.95–1.69 in Model 2). After adjusting for age, sex, and area, there was no significant difference between *COBLL1* rs6717858 genotypes and BMI in the additive model (Table S3).

### 2.5. Association between COBLL1 rs6717858 Genotypes, Dietary Fat Intake, and Incidence of Obesity

Table 6 shows the association between the *COBLL1* rs6717858 genotypes and incidence of obesity, stratified by tertiles of dietary fat. In Model 1, men in tertile 3 of dietary fat with the CT or CC genotype had a 66% higher incidence of obesity than that of men in tertile 1 with the TT genotype (95% CI: 1.07–2.58, *p*-interaction = 0.52). Women in tertile 3 of dietary fat with the TT genotype had a 49% higher incidence of obesity than that of women in tertile 1 with the TT genotype (95% CI: 1.08–2.06, *p*-interaction = 0.09). In Model 2, men in tertile 3 of dietary fat with the CT or CC genotype had a 63% higher incidence of obesity than that of men in tertile 1 with the TT genotype (HR: 1.63, 95% CI: 1.04–2.56, *p*-interaction = 0.49). Women in tertile 3 of dietary fat with the TT genotype had a 53% higher incidence of obesity than that of women in tertile 1 with the TT genotype (95% CI: 1.10–2.13, *p*-interaction = 0.08). 

We further investigated the association between *COBLL1* rs6717858 genotypes and incidence of obesity, stratified by dietary fat intake (<15% vs. ≥15%; Table S4). In men, there was no association between *COBLL1* rs6717858 genotypes and obesity, stratified by dietary fat. In Model 1, women with the TT genotype had a 31% higher incidence of obesity than that of women in the <15% group (≥15%: 95% CI: 1.01–1.70, *p*-interaction = 0.03). Women with the CT or CC genotype had a 64% higher incidence of obesity than that of women with TT genotype in the <15% group (< 15%: 95% CI: 1.16–2.30, *p*-interaction = 0.03). In Model 2, women with the TT genotype had a 34% higher incidence of obesity than that of women in the <15% group (≥15%: 95% CI: 1.02–1.75, *p*-interaction = 0.02). Women with the CT or CC genotype had a 64% higher incidence of obesity than that of women with the TT genotype in the <15% group (<15%: 95% CI: 1.17–2.31, *p*-interaction = 0.02).

## 3. Discussion

Using large-scale prospective cohort data from Ansan and Ansung, we identified an interaction between *COBLL1* genetic variants and dietary fat intake in obese patients. After adjusting for covariates, men in the highest tertile of dietary fat intake with the CT or CC genotype had 63% increased incidence of obesity compared to those men in the lowest tertile of dietary fat intake with the TT genotype. In women, those in the highest tertile of dietary fat intake with the TT genotype had 53% increased incidence of obesity compared to those in the lowest tertile of dietary fat intake with the TT genotype. 

The association between the *COBLL1* rs6717858 genotypes and the incidence of obesity based on sex was not significant. The *COBLL1* gene is associated with abdominal obesity, BMI, fat mass, WC, waist-to-hip ratio, and blood lipids [26,27,34,35], and influences obesity risk by inhibiting lipid storage in adipose tissue [36]. The current study examined middle-aged Korean adults, whereas a genetic study of European and European-American populations revealed a negative association between *GRB14/COBLL1* rs6717858 (T allele) and BMI, weight, and BMI-adjusted WC [40]. In addition, the previous study included 120,975 men and 142,332 women [40], in contrast to our study, which included only 1540 men and 1515 women. Therefore, differences in race and number of study participants may have influenced the results.

Men in the highest tertile of dietary fat had a 41% higher incidence of obesity compared to men in the lowest tertile of dietary fat. In women, there was no significant difference in obesity regardless of the adjusted covariates. The association between dietary fat and obesity was stronger in men because the rate of lipoprotein metabolism for dietary fat is lowered owing to the androgen-inhibitory effect [41]. These findings are consistent with those of previous studies indicating that dietary fat increases the risk of obesity [42,43,44,45,46,47,48]. In several experimental animal studies, a HF diet was found to increase energy intake, body weight, and body fat [42,43,44]. A calorie-limited HF diet decreased the weight of 16-week-old C57BL/6J mice during the early stages but caused rebound weight gain later [44]. The fat intake was positively linked to BMI in 3484 Chinese adults aged 20–45 years [46]. The high ratio of dietary fat resulted in weight gain in 41,518 American women aged 30–55 years [47]. The high intake of animal, saturated, and trans fats resulted in higher weight gain [48]. 

Fat intake also increases the risk of obesity by altering several hormones, such as serotonin and leptin [42,45]. Dietary fat interferes with hypothalamic neurotransmission by decreasing serotonin levels, which is crucial for controlling energy homeostasis and leading to weight gain [42]. A 27-week HF diet increased the weight, energy intake, and adipocyte size with hyperleptinemia risk in 14–16-week-old C57BL/6J mice [45]. Fat intake increases the risk of obesity by decreasing serotonin and increasing leptin, leptin resistance, adipose tissue, fat mass, body weight, and energy intake [42,43,44,45,46,47,48].

Our findings showed that the influence of fat intake on the incidence of obesity differs according to the *COBLL1* rs6717858 genotypes and sex. Fat intake in men with the CT or CC genotype and women with the TT genotype was positively associated with obesity, independent of the adjusted covariates. Fat intake in tertile 2 was positively associated with obesity in women with the CT or CC genotype, independent of the adjusted covariates. Among the women with the CT or CC genotype, there were 60 cases in total (tertile 1: 30/121, tertile 2: 22/78, and tertile 3: 8/63). The small number of women may have a significant effect on the lack of statistical tests.

To improve public health and prevent chronic diseases, the Korean Nutrition Society recommends an appropriate fat intake of 15–30% in Korean adults [49]. In terms of the percentage of energy from dietary fat (< 15% vs. ≥ 15%), our study also shows that the incidence of obesity differs according to the *COBLL1* rs6717858 genotypes and sex. There were no significant differences in obesity among the men. In women with the TT genotype, a fat intake of ≥ 15% increased the incidence of obesity. In women with the CT or CC genotype, a fat intake of < 15% increased the incidence of obesity. Fat intake has a different effect on obesity, depending on sex and genetic variants. Some studies have indicated that fat intake can prevent obesity [50,51]. In a prospective cohort study, HF dairy intake was negatively associated with obesity in 3157 American adults aged 18–30 years [50]. In another prospective cohort study, the substitution of monounsaturated or polyunsaturated fatty acids with saturated fatty acids resulted in weight loss in 6942 Spanish adults [51].

Controlling leptin levels by *COBLL1* gene expression contributes to the influence of a HF diet in obesity [39,52,53,54]. Increased leptin resistance due to dietary fat intake affects obesity [52,53,54]. Dietary fat intake increases in leptin resistance as a result of chronic increase in leptin levels, which hinders leptin signaling to satiety via hypothalamic inflammation [52,53]. Leptin resistance increases food intake owing to loss of leptin function, which promotes obesity [54]. Dietary fat intake is associated with leptin levels in adipose tissues through *COBLL1* gene expression [39]. In a genome-wide meta-analysis of leptin-level-related genes, a HF diet was linked to higher *COBLL1* gene expression in adipocytes in 4-month-old mice [39].

In this study, the incidence of obesity for the *COBLL1* rs6717858 genotypes differed according to sex, suggesting an effect on sex hormones. The major T allele of *COBLL1* rs6717858 is associated with abdominal obesity, body fat, WC, and blood lipid accumulation, especially in women [26,27,34,35]. In men, low levels of gonadal androgen and adrenal C19 steroids contribute to obesity [55]. Menopause-related estrogen deficiency causes abdominal obesity, which increases CVD risk in menopausal women [55]. Sex hormone secretion varies between men and women; therefore, hormonal effects on the function and deposition of adipose tissue will also differ [56]. As this study was conducted in middle-aged adults, changes in sex hormones among the participants may have affected obesity [56,57].

This study had certain strengths. First, to our knowledge, it was the first to investigate the interaction between *COBLL1* rs6717858 genotypes and dietary fat in obesity based on sex in a follow-up study. Second, the causal association between obesity and gene–nutrient factor was clear because of the prospective cohort study design. Third, several covariates that may interfere with obtaining accurate results were adjusted for. Fourth, the effects of basic covariates (including socioeconomic indicators, alcohol consumption, smoking, MET, and BMI) on incidence of obesity, and the effects of these factors after adjusting for dietary variables, were taken into account.

This study also had some limitations. First, the number of women with the CT or CC genotype (minor C allele) was small, which may have affected the statistical power. Second, because this study was conducted among Koreans, the findings cannot be generalized to other races/ethnicities. Third, obesity is common regardless of age; however, this study focused on middle-aged adults. Fourth, because only dietary data at baseline were used during analysis, dietary changes over time should be considered.

Based on these limitations, future research directions must consider three perspectives. First, because different types of dietary fat have different effects in obesity, the influence of dietary fat type on the incidence of obesity requires further investigation. Second, because obesity occurs regardless of age, its incidence should be studied across all age groups, not only among middle-aged individuals. Third, the ratio of various nutrients to total energy intake will determine the influence of dietary fat in obesity.

In conclusion, we present new information on the interaction between dietary fat and *COBLL1* in obesity among middle-aged Korean adults. Dietary fat is positively associated with obesity in men. An important finding was that dietary fat was positively associated with obesity in men with the CT or CC genotype independent of the adjusted covariates. Dietary fat intake in women with the TT genotype was positively associated with obesity independent of the adjusted covariates. These results suggest that fat intake in obesity differs according to sex and genetic variants. Our results highlight the importance of environmental factors considering individual genotypes and could help to reduce obesity using basic scientific data for personalized nutrition.

## 4. Materials and Methods

### 4.1. Data Source and Study Participants

This study used data from the Korean Genome and Epidemiology Study (KoGES) and included a total of 3055 Korean adults (1540 men and 1515 women). To identify potential risks for common diseases, KoGES, a large population-based prospective cohort study, has recruited participants since 2001 and conducted follow-up surveys on occurrence of new diseases and lifestyle changes. Participants were followed up every 2 years to determine the association between genetic and environmental factors. This study included KoGES data from baseline (2001–2002) to follow-up (2003–2014). The study protocol was examined and approved by the Institutional Review Board (IRB) of Inha University on 31 January 2020 (IRB No. 200129–1A).

**Figure 1:**
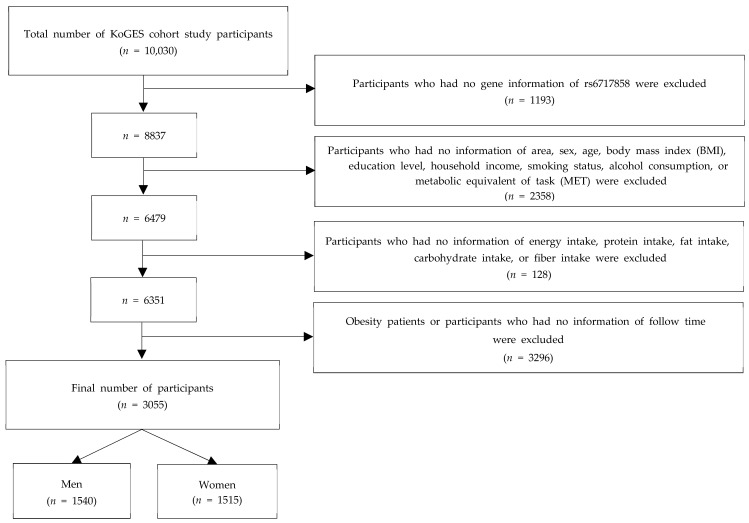
Process flow diagram outlining the steps involved in the recruitment process.

The participants included 10,030 adults, 5018 of whom were recruited from an urban area in Ansan and 5012 from an agricultural area in Ansung. At baseline, we excluded those who had no single nucleotide polymorphism (SNP) data (*n* = 1193), no information of area, sex, age, BMI, alcohol consumption, smoking status, metabolic equivalent of task (MET) (*n* = 2358), total energy, carbohydrate, protein, fat, or fiber intake (*n* = 128), obesity or observation period (*n* = 3296). Finally, this study included 3055 participants (1540 men and 1515 women) (Figure 1).

### 4.2. Dietary Assessment

A semi-quantitative food-frequency questionnaire (SQ-FFQ) was used to obtain dietary information at baseline. The SQ-FFQ was used to evaluate the amount and frequency of food consumption by Koreans aged 40–69 years in the past year. The SQ-FFQ excluded those with a total energy intake of <100 kcal or >10,000 kcal of energy per day. Intake of fatty foods was calculated by multiplying the nutrient content of each food unit. Dietary fat intake (g/1000 kcal) was assessed as fat (g/day)/total energy (kcal) × 1000. The ratio of dietary fat intake (kcal/day) was calculated as fat (g/day) × 9/total energy (kcal) × 100 and classified into tertiles according to dietary fat (lowest, medium, and highest). According to the Korean Nutrition Society in 2020, the recommended range of dietary fat intake (% energy) was 15–30% for Koreans [49]. The association between *COBLL1* rs6717858 genotypes and the incidence of obesity was classified into two groups: <15% and ≥15%. Few study participants consumed >30% fat (9 men and 14 women); therefore, groups with ≥15% fat intake were combined.

### 4.3. Definition of Obesity

The BMI for diagnosing obesity was calculated as kg/m^2^. Height was measured with the participants standing on a horizontal surface while wearing light clothing, with the heel, buttocks, back, and head bordered by a vertical plate and the head facing forward. The measured height was read to one decimal place in centimeters. Body weight was measured with the participants wearing minimal clothing and standing on a flat floor. The measured weight was read to the nearest 10 g. Obesity was assessed as BMI ≥ 25 kg/m^2^ using World Health Organization standards for Asians [58]. In this study, only the second to the seventh obese patients were targeted, and obese patients at baseline were excluded.

### 4.4. Genotyping and Imputation

Korean genome data were obtained using the genome-wide human SNP Array 5.0 from Affymetrix, which is commonly used in many studies on the genomes of various diseases [59,60,61]. We selected the *COBLL1* gene (rs6717858) because it has previously been linked to blood lipid accumulation, plasma leptin levels, central obesity, body fat, BMI, and WC [26,27,33,34,35]. Markers were included as criteria for a call rate ≥ 95%, INFO ≥ 0.8, and Hardy–Weinberg equilibrium *p* ≥ 1.0 × 10^−6^ based on standard quality control procedures [62].

### 4.5. Statistical Analyses

Genetic analysis of *COBLL1* rs6717858 (minor allele, C) was conducted using PLINK (version 1.90 beta). Dietary fat intake was divided according to sex into tertiles, medians, and continuous types. The association between dietary fat (g/1000 kcal) and BMI was also analyzed. The association between *COBLL1* rs6717858 genotypes and BMI was evaluated using linear regression analysis and presented as beta and SE. The general characteristics of obesity (obese vs. non-obese) and *COBLL1* rs6717858 genotypes (TT vs. CT or CC) were compared using *t*-tests for continuous variables and chi-squared tests for categorical variables. The HRs and 95% CIs for the interactions between *COBLL1* rs6717858 genotypes and dietary fat intake in obesity were calculated using multivariable Cox proportional hazard models. Model 1 included the following variables: age, area, education level, income level, alcohol consumption, smoking status, MET, and BMI. Model 2 further included the following variables: total energy and dietary fiber. All statistical analyses were performed using SAS software (version 9.4; SAS Institute, Cary, NC, USA). The significance level was set at *p* < 0.05.

## Figures and Tables

**Table 1:** General characteristics, biomarkers, and dietary intake of study participants based on sex and obesity status.

Variables	Men (*n* = 1540)			Women (*n* = 1515)		
	Non-Obese	Obese	*p*-Value ^(1)^	Non-Obese	Obese	*p*-Value ^(1)^
Participants, *n*	1228	312		1200	315	
rs6717858 genotypes			0.34			0.36
TT (*n* = 2473)	979 (79.7%)	241 (77.2%)		998 (83.2%)	255 (81.0%)	
CT, CC (*n* = 582)	249 (20.3%)	71 (22.8%)		202 (16.8%)	60 (19.1%)	
Age (years)	51.8 ± 8.9	48.9 ± 7.6	<0.0001	50.7 ± 8.9	50.9 ± 8.6	0.66
Area			0.002			0.57
Ansung	419 (34.1%)	72 (23.1%)		444 (37.0%)	122 (38.7%)	
Ansan	809 (65.9%)	240 (76.9%)		756 (63.0%)	193 (61.3%)	
Education level			0.84			0.88
College or lower	1040 (84.7%)	260 (83.3%)		1126 (93.8%)	295 (93.7%)	
University	156 (12.7%)	43 (13.8%)		69 (5.8%)	18 (5.7%)	
Graduate school or higher	32 (2.6%)	9 (2.9%)		5 (0.4%)	2 (0.6%)	
Household income (1 million won/month)			0.0035			0.2
<1	336 (27.4%)	64 (20.5%)		388 (32.3%)	117 (37.2%)	
1–3	624 (50.8%)	155 (49.7%)		594 (49.5%)	139 (44.1%)	
>3	268 (21.8%)	93 (29.8%)		218 (18.2%)	59 (18.7%)	
BMI (kg/m²)	22.1 ± 1.8	24.0 ± 0.8	<0.0001	22.2 ± 1.7	23.9 ± 1.0	<0.0001
Alcohol consumption			0.0035			0.4
None	336 (27.4%)	64 (20.5%)		850 (70.8%)	219 (69.5%)	
Past	624 (50.8%)	155 (49.7%)		32 (2.7%)	13 (4.1%)	
Current	268 (21.8%)	93 (29.8%)		318 (26.5%)	83 (26.4%)	
Smoking status			0.16			0.96
None	240 (19.5%)	61 (19.6%)		1149 (95.8%)	301 (95.6%)	
Past	121 (9.9%)	20 (6.4%)		13 (1.1%)	4 (1.3%)	
Current	867 (70.6%)	231 (74.0%)		38 (3.1%)	10 (3.1%)	
MET (hours/day) ^(2)^	170.6 ± 106.6	156.0 ± 97.1	0.02	151.5 ± 92.6	157.4 ± 96.2	0.32
Biomarkers						
Waist circumference (cm)	78.5 ± 5.8	82.6 ± 4.2	<0.0001	74.8 ± 7.1	78.8 ± 7.2	<0.0001
HDL-cholesterol (mg/dL) ^(3)^	45.7 ± 10.3	43.8 ± 10.2	0.003	47.7 ± 10.3	46.8 ± 10.6	0.17
Triglyceride (mg/dL)	155.1 ± 109.8	173.3 ± 99.8	0.0048	129.2 ± 71.73	140.1 ± 101.3	0.07
Fasting glucose (mg/dL)	88.9 ± 21.8	89.0 ± 29.9	0.96	82.4 ± 14.7	83.5 ± 21.1	0.39
Blood pressure						
Average systolic blood pressure (mmHg)	119.4 ± 16.6	118.8 ± 16.5	0.62	115.0 ± 18.4	116.2 ± 17.8	0.27
Average diastolic blood pressure (mmHg)	79.7 ± 10.69	80.24 ± 10.52	0.42	75.0 ± 11.3	76.4 ± 10.8	0.04
Dietary intake						
Total energy intake (kcal/day)	1979 ± 609	2070 ± 619	0.02	1886 ± 696	1876 ± 682	0.83
Protein intake (% energy)	13.6 ± 2.3	13.9 ± 2.1	0.0069	13.5 ± 2.4	13.5 ± 2.2	0.72
Fat intake (% energy)	15.4 ± 5.1	16.4 ± 4.8	0.001	14.0 ± 5.4	14.1 ± 5.1	0.78
Carbohydrate intake (% energy)	69.8 ± 6.6	68.5 ± 6.1	0.001	71.5 ± 7.1	71.4 ± 6.6	0.86
Fiber intake (g/day)	6.7 ± 3.0	6.9 ± 3.6	0.46	6.9 ± 3.3	7.2 ± 4.3	0.23

Data are presented as the mean ± standard deviation (SD) or number (%). ^(1)^ *p*-values were calculated using the chi-squared test for categorical variables and *t*-test for continuous variables. ^(2)^ MET, metabolic equivalent of task. ^(3)^ HDL-cholesterol, high-density lipoprotein cholesterol.

**Table 2:** General characteristics, biomarkers, and dietary intake of study participants based on sex and *COBLL1* rs6717858 genotypes.

Variables	Men (*n* = 1540)			Women (*n* = 1515)		
	*COBLL1* rs6717858 Genotypes					
	TT	CT, CC	*p*-Value ^(1)^	TT	CT, CC	*p*-Value ^(1)^
Participants, *n*	1220	320		1253	262	
Age (years)	51.3 ± 8.7	50.8 ± 8.7	0.33	50.4 ± 8.8	52.0 ± 9.1	0.0082
Area			0.34			0.39
Ansung	396 (32.5%)	95 (29.7%)		462 (36.9%)	104 (39.7%)	
Ansan	824 (67.5%)	225 (70.3%)		791 (63.1%)	158 (60.3%)	
Education level			0.2			0.32
College or lower	1033 (84.7%)	267 (83.4%)		1171 (93.5%)	250 (95.4%)	
University	151 (12.3%)	48 (15.0%)		75 (6.0%)	12 (4.6%)	
Graduate school or higher	36 (3.0%)	5 (1.6%)		7 (0.5%)	0 (0%)	
Household income (1 million won/month)			0.91			0.68
<1	320 (26.2%)	80 (25.0%)		417 (33.3%)	88 (33.6%)	
1–3	615 (50.4%)	164 (51.2%)		602 (48.0%)	131 (50.0%)	
>3	285 (23.4%)	76 (23.8%)		234 (18.7%)	43 (16.4%)	
Body mass index (kg/m²)	22.5 ± 1.8	22.6 ± 1.8	0.42	22.5 ± 1.7	22.4 ± 1.8	0.35
Alcohol consumption			0.4			0.16
None	247 (20.2%)	54 (16.9%)		889 (71.0%)	180 (68.7%)	
Past	111 (9.1%)	30 (9.3%)		41 (3.2%)	4 (1.5%)	
Current	862 (70.7%)	236 (73.8%)		323 (25.8%)	78 (29.8%)	
Smoking status			0.54			0.17
None	242 (19.8%)	72 (22.5%)		1201 (95.9%)	249 (95.0%)	
Past	370 (30.4%)	91 (28.4%)		16 (1.2%)	1 (0.4%)	
Current	608 (49.8%)	157 (49.1%)		36 (2.9%)	12 (4.6%)	
MET (hours/day) ^(2)^	168.2 ± 105.4	167.1 ± 102.5	0.88	151.6 ± 93.6	157.9 ± 92.3	0.33
Waist circumference (cm)	79.3 ± 5.7	79.2 ± 5.8	0.77	75.6 ± 7.2	75.7 ± 7.6	0.78
HDL-cholesterol (mg/dL) ^(3)^	45.0 ± 10.2	46.5 ± 10.6	0.03	47.6 ± 10.3	47.0 ± 10.4	0.41
Triglyceride (mg/dL)	162.0 ± 114.9	145.8 ± 76.2	0.0027	129.7 ± 77.8	140.1 ± 83.7	0.05
Fasting glucose (mg/dL)	89.0 ± 21.9	89.0 ± 29.5	0.96	82.7 ± 16.8	82.0 ± 13.3	0.46
Blood pressure						
Average systolic blood pressure (mmHg)	119.1 ± 16.3	119.8 ± 17.8	0.56	114.7 ± 18.0	117.8 ± 19.4	0.01
Average diastolic blood pressure (mmHg)	79.8 ± 10.6	79.7 ± 11.0	0.83	74.9 ± 11.0	76.9 ± 12.1	0.01
Dietary intake						
Total energy intake (kcal/day)	1998 ± 595	1993 ± 676	0.89	1890 ± 674	1854 ± 777	0.48
Protein intake (% energy)	13.5 ± 2.4	13.5 ± 2.2	0.72	13.5 ± 2.3	13.4 ± 2.5	0.42
Fat intake (% energy)	15.6 ± 5.1	15.7 ± 4.8	0.54	14.2 ± 5.3	13.5 ± 5.5	0.04
Carbohydrate intake (% energy)	69.6 ± 6.6	69.3 ± 6.2	0.37	71.3 ± 6.9	72.1 ± 7.2	0.08
Fiber intake (g/day)	6.7 ± 3.0	6.9 ± 3.7	0.41	7.0 ± 3.5	7.0 ± 3.9	0.98

Data are presented as the mean ± standard deviation (SD) or number (%). ^(1)^ *p*-values were calculated using the chi-squared test for categorical variables and *t*-test for continuous variables. ^(2)^ MET, metabolic equivalent of task. ^(3)^ HDL-cholesterol, high-density lipoprotein cholesterol.

**Table 3:** Association between dietary fat intake and BMI.

		Men (*n* = 1540)		Women (*n* = 1515)	
		BMI (kg/m^2^)			
		Beta ± SE	*p*-Value	Beta ± SE	*p*-Value
Dietary fat (g/1000 kcal)	Model 1 ^(1)^	−0.001 ± 0.009	0.89	−0.004 ± 0.008	0.58
	Model 2 ^(2)^	−0.015 ± 0.009	0.12	−0.003 ± 0.008	0.71

SE, standard error; BMI, body mass index. ^(1)^ Adjusted for age, area, alcohol consumption, smoking, BMI, education level, household income, and metabolic equivalent of task (MET). ^(2)^ Adjusted for age, area, alcohol consumption, smoking, BMI, education level, household income, MET, total energy intake, and dietary fiber intake.

**Table 4:** Association between tertiles of dietary fat intake and incidence of obesity.

	Dietary Fat (% Energy)					
**Men (*n* = 1540)**	**Tertile 1**		**Tertile 2**		**Tertile 3**	
Median (ranges)	10.7 (2.9–13.2)		15.4 (13.2–17.5)		20.3 (17.5–35.1)	
Person-years	4717.5		4653.1		4634.0	
Incident cases (*n*)	84/513		109/514		119/513	
	HR (95% CI)	*p*-value	HR (95% CI)	*p*-value	HR (95% CI)	*p*-value
Model 1 ^(1)^	1.00 (Ref.)		1.13 (0.81–1.57)	0.46	1.41 (1.02–1.95)	0.04
Model 2 ^(2)^	1.00 (Ref.)		1.13 (0.81–1.57)	0.48	1.37 (0.98–1.93)	0.07
**Women (*n* = 1515)**	**Tertile 1**		**Tertile 2**		**Tertile 3**	
Median (ranges)	8.9 (1.9–11.5)		13.7 (11.5–15.9)		18.9 (15.9–42.0)	
Person-years	4582.6		4622.1		4753.4	
Incident cases (*n*)	97/505		108/505		110/505	
	HR (95% CI)	*p*-value	HR (95% CI)	*p*-value	HR (95% CI)	*p*-value
Model 1 ^(1)^	1.00 (Ref.)		0.96 (0.72–1.28)	0.79	1.31 (0.97–1.78)	0.08
Model 2 ^(2)^	1.00 (Ref.)		0.97 (0.72–1.29)	0.81	1.35 (0.99–1.84)	0.06

HR, hazard ratio; CI, confidence interval; Ref., reference. ^(1)^ Adjusted for age, sex, area, alcohol consumption, smoking, body mass index (BMI), education level, household income, metabolic equivalent of task (MET). ^(2)^ Adjusted for age, sex, area, alcohol consumption, smoking, BMI, education level, household income, MET, total energy, and dietary fiber.

**Table 5:** Association between *COBLL1* rs6717858 genotypes and incidence of obesity.

	*COBLL1* rs6717858 Genotypes		
**Men (*n* = 1540)**	**TT**	**CT, CC**	
	HR (95% CI)	HR (95% CI)	*p*-value
Model 1 ^(1)^	1.00 (Ref.)	1.08 (0.83–1.41)	0.58
Model 2 ^(2)^	1.00 (Ref.)	1.08 (0.83–1.41)	0.58
**Women (*n* = 1515)**	**TT**	**CT, CC**	
	HR (95% CI)	HR (95% CI)	*p*-value
Model 1 ^(1)^	1.00 (Ref.)	1.29 (0.97–1.71)	0.09
Model 2 ^(2)^	1.00 (Ref.)	1.27 (0.95–1.69)	0.10

HR, hazard ratio; CI, confidence interval; Ref., reference. ^(1)^ Adjusted for age, sex, area, alcohol consumption, smoking, body mass index (BMI), education level, household income, and metabolic equivalent of task (MET). ^(2)^ Adjusted for age, sex, area, alcohol consumption, smoking, BMI, education level, household income, MET, total energy, and dietary fiber.

**Table 6:** Association between *COBLL1* rs6717858 genotypes and incidence of obesity, stratified by tertiles of dietary fat intake.

		Men (*n* = 1540)							Women (*n* = 1515)						
		Dietary Fat (% energy)													
		Tertile 1		Tertile 2		Tertile 3		*p*-Interaction	Tertile 1		Tertile 2		Tertile 3		*p*-Interaction
Median		10.7		15.4		20.3			8.9		13.7		18.9		
		HR (95% CI)	*p*-value	HR (95% CI)	*p*-value	HR (95% CI)	*p*-value		HR (95% CI)	*p*-value	HR (95% CI)	*p*-value	HR (95% CI)	*p*-value	
Model 1 ^(1)^	TT	1.00 (Ref.)		1.19 (0.82–1.71)	0.36	1.37 (0.95–1.96)	0.09	0.52	1.00 (Ref.)		1.43 (0.95–2.17)	0.62	1.49 (1.08–2.06)	0.02	0.09
	CT, CC	1.08 (0.54–2.15)	0.82	1.03 (0.62–1.70)	0.91	1.66 (1.07–2.58)	0.03		1.50 (0.95–2.37)	0.09	1.68 (1.04–2.72)	0.03	0.90 (0.43–1.88)	0.78	
Model 2 ^(2)^	TT	1.00 (Ref.)		1.18 (0.82–1.71)	0.37	1.32 (0.91–1.92)	0.15	0.49	1.00 (Ref.)		0.93 (0.67–1.29)	0.67	1.53 (1.10–2.13)	0.01	0.08
	CT, CC	1.09 (0.55–2.17)	0.80	1.01 (0.61–1.68)	0.96	1.63 (1.04–2.56)	0.03		1.44 (0.95–2.18)	0.09	1.63 (1.01–2.64)	0.0458	0.92 (0.44–1.94)	0.84	

HR, hazard ratio; CI, confidence interval; Ref., reference. ^(1)^ Adjusted for age, sex, area, alcohol consumption, smoking, body mass index (BMI), education level, household income, metabolic equivalent of task (MET). ^(2)^ Adjusted for age, sex, area, alcohol consumption, smoking, BMI, education level, household income, MET, total energy, and dietary fiber.

## Data Availability

The data underlying the results of our study are not publicly available because of the KoGES data policy. Data are available from the Division of Genetic Epidemiology and Health Index, NIH, Korea Centers for Disease Control and Prevention for researchers who meet the criteria for access to confidential data.

## Supplementary Materials

The following supporting information can be downloaded at: https://www.mdpi.com/article/10.3390/ijms24043758/s1.

## References

[1] Blüher M. (2019). Obesity: Global epidemiology and pathogenesis. Nat. Rev. Endocrinol..

[2] Lee G.B., Kim Y., Park S., Kim H.C., Oh K. (2022). Obesity, hypertension, diabetes mellitus, and hypercholesterolemia in Korean adults before and during the COVID-19 pandemic: A special report of the 2020 Korea National Health and Nutrition Examination Survey. Epidemiol. Health.

[3] Magalhaes C., Carvalho M., Sousa L., Caramelli P., Gomes K. (2015). Leptin in Alzheimer’s disease. Anal. Chim. Acta.

[4] Hoong C.W.S., Hussain I., Aravamudan V.M., Phyu E.E., Lin J.H.X., Koh H. (2021). Obesity is associated with poor COVID-19 outcomes: A systematic review and meta-analysis. Horm. Metab. Res..

[5] Pietri L., Giorgi R., Bégu A., Lojou M., Koubi M., Cauchois R., Grangeot R., Dubois N., Kaplanski G., Valéro R. (2021). Excess body weight is an independent risk factor for severe forms of COVID-19. Metabolism.

[6] Herbert A., Gerry N.P., McQueen M.B., Heid I.M., Pfeufer A., Illig T., Wichmann H.E., Meitinger T., Hunter D., Hu F.B. (2006). A common genetic variant is associated with adult and childhood obesity. Science.

[7] Farooqi I.S., Wangensteen T., Collins S., Kimber W., Matarese G., Keogh J.M., Lank E., Bottomley B., Lopez Fernandez J., Ferraz Amaro I. (2007). Clinical and molecular genetic spectrum of congenital deficiency of the leptin receptor. N. Engl. J. Med..

[8] Licinio J., Caglayan S., Ozata M., Yildiz B.O., De Miranda P.B., O’Kirwan F., Whitby R., Liang L., Cohen P., Bhasin S. (2004). Phenotypic effects of leptin replacement on morbid obesity, diabetes mellitus, hypogonadism, and behavior in leptin-deficient adults. Proc. Natl. Acad. Sci. USA.

[9] Farooqi I.S., Matarese G., Lord G.M., Keogh J.M., Lawrence E., Agwu C., Sanna V., Jebb S.A., Perna F., Fontana S. (2002). Beneficial effects of leptin on obesity, T cell hyporesponsiveness, and neuroendocrine/metabolic dysfunction of human congenital leptin deficiency. J. Clin. Investig..

[10] Bray G.A., Popkin B.M. (1998). Dietary fat intake does affect obesity!. Am. J. Clin. Nutr..

[11] Wang L., Wang H., Zhang B., Popkin B.M., Du S. (2020). Elevated fat intake increases body weight and the risk of overweight and obesity among Chinese adults: 1991–2015 trends. Nutrients.

[12] Hall K.D., Bemis T., Brychta R., Chen K.Y., Courville A., Crayner E.J., Goodwin S., Guo J., Howard L., Knuth N.D. (2015). Calorie for calorie, dietary fat restriction results in more body fat loss than carbohydrate restriction in people with obesity. Cell Metab..

[13] Cani P.D., Bibiloni R., Knauf C., Waget A., Neyrinck A.M., Delzenne N.M., Burcelin R. (2008). Changes in gut microbiota control metabolic endotoxemia-induced inflammation in high-fat diet–induced obesity and diabetes in mice. Diabetes.

[14] Machate D.J., Figueiredo P.S., Marcelino G., Guimarães R.d.C.A., Hiane P.A., Bogo D., Pinheiro V.A.Z., Oliveira L.C.S.d., Pott A. (2020). Fatty acid diets: Regulation of gut microbiota composition and obesity and its related metabolic dysbiosis. Int. J. Mol. Sci..

[15] Sun Y., Ge X., Li X., He J., Wei X., Du J., Sun J., Li X., Xun Z., Liu W. (2020). High-fat diet promotes renal injury by inducing oxidative stress and mitochondrial dysfunction. Cell Death Dis.

[16] Maffei á., Halaas J., Ravussin E., Pratley R., Lee G., Zhang Y., Fei H., Kim S., Lallone R., Ranganathan S. (1995). Leptin levels in human and rodent: Measurement of plasma leptin and ob RNA in obese and weight-reduced subjects. Nat. Med..

[17] Hu S., Wang L., Yang D., Li L., Togo J., Wu Y., Liu Q., Li B., Li M., Wang G. (2018). Dietary fat, but not protein or carbohydrate, regulates energy intake and causes adiposity in mice. Cell Metab..

[18] Wang H., Storlien L.H., Huang X.F. (2002). Effects of dietary fat types on body fatness, leptin, and ARC leptin receptor, NPY, and AgRP mRNA expression. Am. J. Physiol. Endocrinol. Metab..

[19] Huang L., Li C. (2000). Leptin: A multifunctional hormone. Cell Res..

[20] Zhang Y., Scarpace P.J. (2006). The role of leptin in leptin resistance and obesity. Physiol. Behav..

[21] Simon M.S., Heilbrun L.K., Boomer A., Kresge C., Depper J., Kim P.N., Valeriote F., Martino S. (1997). A randomized trial of a low-fat dietary intervention in women at high risk for breast cancer. Nutr. Cancer..

[22] Jeffery R.W., Hellerstedt W.L., French S.A., Baxter J.E. (1995). A randomized trial of counseling for fat restriction versus calorie restriction in the treatment of obesity. Int. J. Obes. Relat. Metab. Disord..

[23] Carroll E.A., Gerrelli D., Gasca S., Berg E., Beier D.R., Copp A.J., Klingensmith J. (2003). Cordon-bleu is a conserved gene involved in neural tube formation. Dev. Biol..

[24] Shungin D., Winkler T.W., Croteau Chonka D.C., Ferreira T., Locke A.E., Mägi R., Strawbridge R.J., Pers T.H., Fischer K., Justice A.E. (2015). New genetic loci link adipose and insulin biology to body fat distribution. Nature.

[25] Scott R.A., Lagou V., Welch R.P., Wheeler E., Montasser M.E., Luan J., Mägi R., Strawbridge R.J., Rehnberg E., Gustafsson S. (2012). Large-scale association analyses identify new loci influencing glycemic traits and provide insight into the underlying biological pathways. Nat. Genet..

[26] Morris A., Voight B., Teslovich T., Wellcome Trust Case Control Consortium, Asian Genetic Epidemiology Network–Type 2 Diabetes (AGEN-T2D) Consortium, South Asian Type 2 Diabetes (SAT2D) Consortium, Diabetes Genetics Replication and Meta-analysis (DIAGRAM) Consortium (2012). Large-scale association analysis provides insights into the genetic architecture and pathophysiology of type 2 diabetes. Nat. Genet..

[27] Teslovich T.M., Musunuru K., Smith A.V., Edmondson A.C., Stylianou I.M., Koseki M., Pirruccello J.P., Ripatti S., Chasman D.I., Willer C.J. (2010). Biological, clinical and population relevance of 95 loci for blood lipids. Nature.

[28] Lu Y., Day F.R., Gustafsson S., Buchkovich M.L., Na J., Bataille V., Cousminer D.L., Dastani Z., Drong A.W., Esko T. (2016). New loci for body fat percentage reveal link between adiposity and cardiometabolic disease risk. Nat. Commun..

[29] Wang Z., Yan Z., Zhang B., Rao Z., Zhang Y., Liu J., Yu L., Zhao Y., Yang B., Wu T. (2013). Identification of a 5-gene signature for clinical and prognostic prediction in gastric cancer patients upon microarray data. Med. Oncol..

[30] Takayama K.i., Suzuki T., Fujimura T., Takahashi S., Inoue S. (2018). COBLL1 modulates cell morphology and facilitates androgen receptor genomic binding in advanced prostate cancer. Proc. Natl. Acad. Sci. USA.

[31] Hussein S., Abdelazem A.S., Abdelmoneem S., Abdelnabi A.S.M., Khamis T., Obaya A.A., Ebian H.F. (2022). Evaluation of miRNA 223/125a and COBLL1 Expressions and ROR-1 Levels as Reliable Markers in B-chronic Lymphocytic Leukemia. Asian Pac. J. Cancer Prev..

[32] Han S., Kim S., Kim H., Lee Y., Choi S., Park G., Kim D., Lee A., Kim J., Choi J. (2017). Cobll1 is linked to drug resistance and blastic transformation in chronic myeloid leukemia. Leukemia.

[33] Goodarzi M.O. (2018). Genetics of obesity: What genetic association studies have taught us about the biology of obesity and its complications. Lancet Diabetes Endocrinol..

[34] Gilks W.P., Abbott J.K., Morrow E.H. (2014). Sex differences in disease genetics: Evidence, evolution, and detection. Trends Genet..

[35] Heid I.M., Jackson A.U., Randall J.C., Winkler T.W., Qi L., Steinthorsdottir V., Thorleifsson G., Zillikens M.C., Speliotes E.K., Mägi R. (2010). Meta-analysis identifies 13 new loci associated with waist-hip ratio and reveals sexual dimorphism in the genetic basis of fat distribution. Nat. Genet..

[36] Chen Z., Yu H., Shi X., Warren C.R., Lotta L.A., Friesen M., Meissner T.B., Langenberg C., Wabitsch M., Wareham N. (2020). Functional screening of candidate causal genes for insulin resistance in human preadipocytes and adipocytes. Circ. Res..

[37] Sun C., Förster F., Gutsmann B., Moulla Y., Stroh C., Dietrich A., Schön M.R., Gärtner D., Lohmann T., Dressler M. (2022). Metabolic Effects of the Waist-To-Hip Ratio Associated Locus GRB14/COBLL1 Are Related to GRB14 Expression in Adipose Tissue. Int. J. Mol. Sci..

[38] Dallner O.S., Marinis J.M., Lu Y.H., Birsoy K., Werner E., Fayzikhodjaeva G., Dill B.D., Molina H., Moscati A., Kutalik Z. (2019). Dysregulation of a long noncoding RNA reduces leptin leading to a leptin-responsive form of obesity. Nat. Med..

[39] Kilpeläinen T.O., Carli J.F.M., Skowronski A.A., Sun Q., Kriebel J., Feitosa M.F., Hedman Å.K., Drong A.W., Hayes J.E., Zhao J. (2016). Genome-wide meta-analysis uncovers novel loci influencing circulating leptin levels. Nat. Commun..

[40] Randall J.C., Winkler T.W., Kutalik Z., Berndt S.I., Jackson A.U., Monda K.L., Kilpeläinen T.O., Esko T., Mägi R., Li S. (2013). Sex-stratified genome-wide association studies including 270,000 individuals show sexual dimorphism in genetic loci for anthropometric traits. PLoS Genet..

[41] Knopp R.H., Paramsothy P., Retzlaff B.M., Fish B., Walden C., Dowdy A., Tsunehara C., Aikawa K., Cheung M.C. (2005). Gender differences in lipoprotein metabolism and dietary response: Basis in hormonal differences and implications for cardiovascular disease. Curr. Atheroscler. Rep..

[42] Haleem D.J., Mahmood K. (2021). Brain serotonin in high-fat diet-induced weight gain, anxiety and spatial memory in rats. Nutr. Neurosci..

[43] Cavaliere G., Viggiano E., Trinchese G., De Filippo C., Messina A., Monda V., Valenzano A., Cincione R.I., Zammit C., Cimmino F. (2018). Long feeding high-fat diet induces hypothalamic oxidative stress and inflammation, and prolonged hypothalamic AMPK activation in rat animal model. Front. Physiol..

[44] McNay D.E., Speakman J.R. (2013). High fat diet causes rebound weight gain. Mol. Metab..

[45] Chakraborty T.R., Donthireddy L., Adhikary D., Chakraborty S. (2016). Long-term high fat diet has a profound effect on body weight, hormone levels, and estrous cycle in mice. Med. Sci. Monit..

[46] Paeratakul S., Popkin B.M., Keyou G., Adair L., Stevens J. (1998). Changes in diet and physical activity affect the body mass index of Chinese adults. Int. J. Obes..

[47] Field A.E., Willett W.C., Lissner L., Colditz G.A. (2007). Dietary fat and weight gain among women in the Nurses’ Health Study. Obesity.

[48] Yang Y., Smith Jr D.L., Keating K.D., Allison D.B., Nagy T.R. (2014). Variations in body weight, food intake and body composition after long-term high-fat diet feeding in C57BL/6J mice. Obesity.

[49] Ministry of Health and Welfare (2020). The Korean Nutrition Society, Dietary Reference Intakes for Koreans. http://www.kns.or.kr/FileRoom/FileRoom_view.asp?idx=108&BoardID=Kdr.

[50] Pereira M.A., Jacobs Jr D.R., Van Horn L., Slattery M.L., Kartashov A.I., Ludwig D.S. (2002). Dairy consumption, obesity, and the insulin resistance syndrome in young adults: The CARDIA Study. JAMA.

[51] Beulen Y., Martínez González M.A., Van de Rest O., Salas Salvadó J., Sorlí J.V., Gómez Gracia E., Fiol M., Estruch R., Santos Lozano J.M., Schröder H. (2018). Quality of dietary fat intake and body weight and obesity in a mediterranean population: Secondary analyses within the PREDIMED trial. Nutrients.

[52] Heshka J.T., Jones P.J. (2001). A role for dietary fat in leptin receptor, OB-Rb, function. Life Sci..

[53] Dragano N.R., Haddad Tovolli R., Velloso L.A. (2017). Leptin, neuroinflammation and obesity. Endocr. Immunol..

[54] Myers M.G., Leibel R.L., Seeley R.J., Schwartz M.W. (2010). Obesity and leptin resistance: Distinguishing cause from effect. Trends Endocrinol. Metab..

[55] Tchernof A., Després J.P. (2000). Sex steroid hormones, sex hormone-binding globulin, and obesity in men and women. Horm. Metab. Res..

[56] Palmer B.F., Clegg D.J. (2015). The sexual dimorphism of obesity. Mol. Cell. Endocrinol..

[57] Field A.E., Colditz G.A., Willett W.C., Longcope C., McKinlay J.B. (1994). The relation of smoking, age, relative weight, and dietary intake to serum adrenal steroids, sex hormones, and sex hormone-binding globulin in middle-aged men. J. Clin. Endocrinol. Metab..

[58] Nuttall F.Q. (2015). Body mass index: Obesity, BMI, and health: A critical review. Nutr. Today.

[59] Hinney A., Nguyen T.T., Scherag A., Friedel S., Brönner G., Müller T.D., Grallert H., Illig T., Wichmann H.E., Rief W. (2007). Genome wide association (GWA) study for early onset extreme obesity supports the role of fat mass and obesity associated gene (FTO) variants. PLoS ONE.

[60] Choi S., Bae S., Park T. (2016). Risk prediction using genome-wide association studies on type 2 diabetes. Genom. Inform..

[61] Lee J.Y., Lee B.S., Shin D.J., Park K.W., Shin Y.A., Kim K.J., Heo L., Lee J.Y., Kim Y.K., Kim Y.J. (2013). A genome-wide association study of a coronary artery disease risk variant. J. Hum. Genet..

[62] Shin D., Lee K.W. (2021). Dietary carbohydrates interact with AMY1 polymorphisms to influence the incidence of type 2 diabetes in Korean adults. Sci. Rep..

