# Supplementary material for: Gene–Nutrient Interactions in Obesity: COBLL1 Genetic Variants Interact with Dietary Fat Intake to Modulate the Incidence of Obesity
Source: Int J Mol Sci. 2023 Feb 13;24(4):3758. doi: 10.3390/ijms24043758 (PMC9959357; doi:10.3390/ijms24043758)
Supplement: Supplementary file 1 [file ijms-24-03758-s001.zip › ijms-2133583-supplementary.pdf]

Table S1. Association between dietary fat intake and incidence of obesity.

Dietary fat	Person-years	Model 1 (1)	Model 2 (2)
		HR (95% CI)	HR (95% CI)
Men (*n* = 1540)			
<15.4 (% energy) (*n* = 769)	7023.1	1.00 (Ref.)	1.00 (Ref.)
≥15.4 (% energy) (*n* = 771)	6981.5	1.23 (0.97-1.55)	1.19 (0.93-1.52)
Continuous (per 1 energy %)	14,004.6	1.03 (1.01-1.05)	1.03 (0.99-1.05)
Women (*n* = 1515)			
<13.7 (% energy) (*n* = 758)	6889.6	1.00 (Ref.)	1.00 (Ref.)
≥13.7 (% energy) (*n* = 757)	7068.5	1.03 (1.01-1.05)	1.03 (0.99-1.05)
Continuous (per 1 energy%)	13,958.1	0.99 (0.99-1.03)	1.01 (0.99-1.03)

HR, hazard ratio; CI, confidence interval; Ref., reference.(1)Adjusted for age, sex, area, alcohol consumption, smoking, body mass index, education level, household income, and metabolic equivalent of task (MET).(2)Adjusted for age, sex, area, alcohol consumption, smoking, body mass index, education level, household income, MET, total energy, and dietary fiber.

Table S2. The incidence of obesity stratified by dietary fat intake (<15% energy, ≥15% energy).

Dietary fat	Men (*n* = 1540)				Women (*n* = 1515)			
(% energy)	< 15%		≥ 15%		< 15%		≥ 15%	
Person-years	6705.5		7299.1		8258.0		5700.1	
Incident cases (*n*)	125/732		187/808		183/909		132/606	
	HR (95% CI)	*p*-value	HR (95% CI)	*p*-value	HR (95% CI)	*p*-value	HR (95% CI)	*p*-value
Model 1 (1)	1.00 (Ref.)		1.27 (1.00-1.61)	0.05	1.00 (Ref.)		1.15 (0.91-1.47)	0.25
Model 2 (2)	1.00 (Ref.)		1.23 (0.96-1.58)	0.11	1.00 (Ref.)		1.17 (0.91-1.49)	0.21

HR, hazard ratio; CI, confidence interval; Ref., reference.(1)Adjusted for age, sex, area, alcohol consumption, smoking, body mass index, education level, household income, metabolic equivalent of task (MET).(2)Adjusted for age, sex, area, alcohol consumption, smoking, body mass index, education level, household income, MET, total energy, and dietary fiber.

Table S3. Association between *COBLL1* rs6717858 genotypes and BMI.

SNP	Chr	Minor allele	BMI (kg/m^2)	
			Beta ± SE	Add *p*-value (1)
COBLL1 rs6717858	2	C	0.12 ± 1.37	0.17

SNP, single nucleotide polymorphism; Chr, chromosome; SE, standard error; Add, additive model; BMI, body mass index.(1)Adjusted for age, sex, and area.

Table S4. Association between *COBLL1* rs6717858 genotypes and incidence of obesity, stratified by dietary fat intake (<15% energy, ≥15% energy).

		Men (*n* = 1540)					Women (*n* = 1515)				
		<15%		≥15%		*p*-interaction	<15%		≥15%		*p*-interaction
		HR (95% CI)	*P*-value	HR (95% CI)	*P*-value		HR (95% CI)	*P*-value	HR (95% CI)	*P*-value	
Model 1 (1)	TT	1.00 (Ref.)		1.22 (0.93-1.61)	0.14	0.63	1.00 (Ref.)		1.31 (1.01-1.70)	0.0458	0.03
	CT, CC	0.99 (0.64-1.53)	0.96	1.39 (0.97-1.99)	0.07		1.64 (1.16-2.30)	0.0046	1.04 (0.60-1.78)	0.90	
Model 2 (2)	TT	1.00 (Ref.)		1.18 (0.89-1.57)	0.25	0.58	1.00 (Ref.)		1.34 (1.02-1.75)	0.03	0.02
	CT, CC	0.98 (0.63-1.52)	0.92	1.35 (0.94-1.95)	0.11		1.64 (1.17-2.31)	0.0046	1.02 (0.59-1.76)	0.93	

HR, hazard ratio; CI, confidence interval; Ref., reference.(1)Adjusted for age, sex, area, alcohol consumption, smoking, body mass index, education level, household income, metabolic equivalent of task (MET).(2)Adjusted for age, sex, area, alcohol consumption, smoking, body mass index, education level, household income, MET, total energy, and dietary fiber.

Table S5. Distribution of dietary fat intake by *COBLL1* rs6717858 genotypes.

	Dietary fat (% energy)		
**Men (*n* = 1540)**	**Tertile 1**	**Tertile 2**	**Tertile 3**
Median (ranges)	10.7 (2.9-13.2)	15.4 (13.2-17.5)	20.3 (17.5-35.1)
Person-years	4717.5	4653.1	4634.0
Incident cases (*n*)	84/513	109/514	119/513
TT	67/416	85/393	89/411
CT, CC	17/97	24/121	30/102
**Women (*n* = 1515)**	**Tertile 1**	**Tertile 2**	**Tertile 3**
Median (ranges)	8.9 (1.9-11.5)	13.7 (11.5-15.9)	18.9 (15.9-42.0)
Person-years	4582.6	4622.1	4753.4
Incident cases (*n*)	97/505	108/505	110/505
TT	72/400	84/431	99/422
CT, CC	25/105	24/74	11/83
